# Case report: Carp edema virus infection in overwintering fish

**DOI:** 10.3389/fvets.2025.1532861

**Published:** 2025-02-14

**Authors:** Miroslava Palikova, Alena Balazova, Lubomir Pojezdal, Ivana Papezikova, Ivana Mikulikova, Ivona Toulova, Jitka Motlova, Jiri Pikula

**Affiliations:** ^1^Department of Ecology and Diseases of Zoo Animals, Game, Fish and Bees, Faculty of Veterinary Hygiene and Ecology, University of Veterinary Science Brno, Brno, Czechia; ^2^Department of Zoology, Fisheries, Hydrobiology and Apiculture, Mendel University in Brno, Brno, Czechia; ^3^Department of Infectious Diseases and Preventive Medicine, Veterinary Research Institute Brno, Brno, Czechia

**Keywords:** aquaculture water temperature, cyprinid fish, emerging viral diseases, poxvirus, susceptibility of carp strains, molecular detection, loop-mediated isothermal amplification

## Abstract

Carp Edema Virus (CEV) has emerged as a viral threat to the sustainability of European pond fisheries, with water temperature and stress playing a crucial role in disease outbreaks. Here, we report on a natural CEV infection in overwintering common carp (*Cyprinus carpio*; *n* = 1,160) broodstock that began to manifest clinically at an unusually low water temperature. In the initial outbreak phase, young broodstock fish exhibited abnormal activity and shoaling at the pond edge. While the water temperature under a discontinuous thin ice layer was 2°C, no deaths were observed. The first fish examined, using standard molecular methods for virological diagnosis, tested negative for CEV. Despite showing clinical signs suggestive of CEV infection, there was no gross pathology except for an increased amount of gill mucus, suggesting that CEV molecular detection may be dependent on infection progression. A shift from a period of cold stress to warming pond water temperatures may have influenced the subsequent progression of the disease. Ongoing clinical signs affected a large part of the population, which remained lethargic and gathered close to the banks. Subsequent virological testing performed ca. 3 weeks after the outbreak and first observation of clinically diseased fish detected the CEV genogroup I agent. CEV-driven die-offs occurred gradually as water temperatures increased to 8°C, with mortalities continuing for ca. 1 month. Interestingly, Přerov scaly carp and Hungarian mirror carp M2 strains differed significantly in mortality rates, at 30 and 60%, respectively. We tested a novel virus detection method, based on loop-mediated isothermal amplification (LAMP) of primers targeting the CEV genogroup I *p4A* gene, for applicability in the field. Samples from moribund fish, cadavers, and pond water all tested positive, with samples positive using LAMP subsequently confirmed by qPCR. To summarize, our data suggest it may be challenging to detect CEV DNA in both the first carp showing signs and surviving carp; scaly and scaleless carp show differential susceptibility to CEV infection; very low water temperatures of 2–4°C permit CEV infection in common carp; the LAMP method is applicable for rapid on-site CEV detection in clinical and environmental samples.

## 1 Introduction

Climate change has had a significant impact on both wild fish and aquacultural stocks through changes in temperature, water quality degradation, habitat loss, and shifts in species distribution (1–3). Such changes not only make fish more susceptible to infection but also enhance the characteristics of pathogens, making them more likely to spread and infect fish populations. An example is Koi Herpesvirus Disease (KHV) in carp fisheries, which is predicted to occur more frequently and persist over longer periods due to changing environmental conditions (4). On the other hand, certain viruses, such as Spring Viraemia of Carp Virus (SVCV), pose a lower risk when water temperatures rise (4). Consequently, the emergence of fish viruses and diseases must be considered an important issue when assessing the health of freshwater aquatic ecosystems and the sustainability of aquacultural fisheries (4).

Ambient temperatures, seasonal variations and stress caused by ongoing climate change are all important factors modulating fish physiological processes, including both innate and acquired immune responses. This, in turn, impacts the development of diseases, including their prevalence, associated morbidity and mortality, or recovery (4–6). While it is known that some pathogenic agents benefit from such changes and others do not (7), it remains difficult to make predictions on the relationship between temperature and fish host-pathogen systems (8). One such example are risk maps displaying geographic regions with more days of water temperatures permissive for virus infection by three important common carp (*Cyprinus carpio*) pathogens, i.e., *Cyvirus cyprinidallo 3* (Koi Herpesvirus), Carp Edema Virus (CEV) and SVCV (9).

Over the past 10 years, infection with CEV, also known as Koi Sleepy Disease, has become a significant threat to European common carp and koi aquaculture (10–17). The combined effects of increasing water temperatures and stressful conditions, such as restocking and fish translocation, play a crucial role in such CEV outbreaks (11, 14, 15). Clinical CEV infections usually occur in common and koi carp at water temperatures of 6–12°C and 15–25°C, respectively (10, 12). Notably, seasonal thermal stratification dynamics in freshwater bodies (18) are also likely to be a factor associated with CEV disease outbreaks.

Here, we report on a case of diagnostic challenge associated with clinical manifestation of CEV in common carp at unusually low water temperatures of 2°C. To our knowledge, this is the lowest water temperature linked to a CEV outbreak. Furthermore, we describe and discuss our findings regarding disease progression in relation to susceptibility differences between scaly and scaleless carp, and the performance of different molecular virological tests for tissue and environmental samples.

## 2 Case presentation and diagnostic assessment

**Figure 1:**
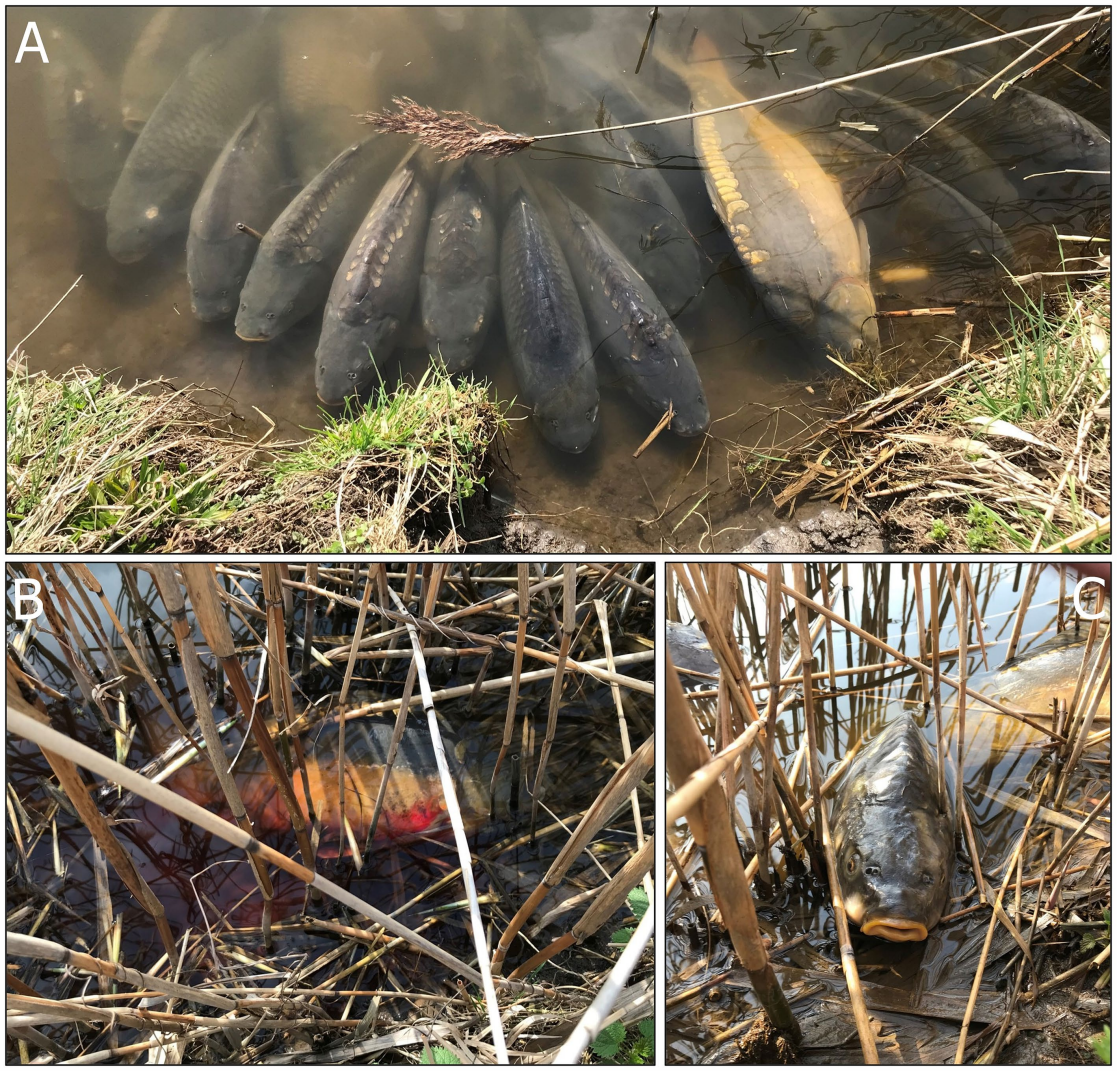
Manifestation of Carp Edema Virus. **(A)** Fish gathered close to the bank exhibiting signs of lethargy, **(B)** a moribund fish bleeding from the gills, **(C)** a dead fish lying on the bank.

**Figure 2:**
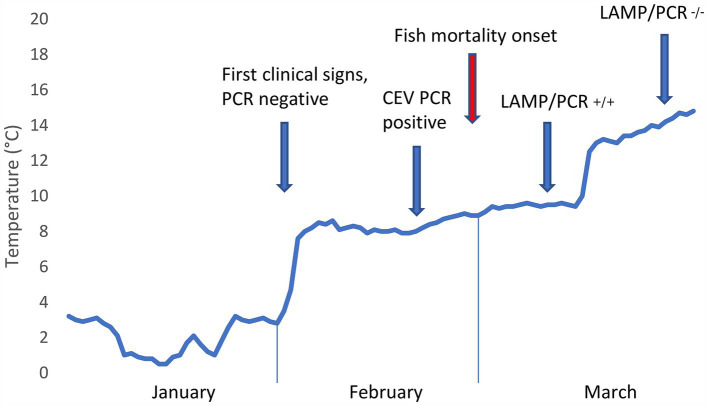
Development of pond water temperature during the winter months of 2024 alongside manifestation of Carp Edema Virus and results of molecular virological tests. Note that the time series of pond water temperature development is based on measurements at the inflow.

The CEV infection was suspected, and later confirmed, at a carp farm in South Moravia, Czech Republic, during the first week of February 2024. The affected fish population consisted of 1,160 broodstock of two strains, Přerov scaly carp (*n* = 460) and Hungarian mirror carp M2 (*n* = 700). The carp, which were being kept in an overwintering pond (0.38 ha, 3,710 m^3^) covered with a thin discontinuous layer of ice, were exhibiting abnormal behavior, such as rising from the bottom and forming shoals near the shoreline (Figure 1A). No mortalities were observed at that time. Water temperature under the ice was 2°C (Figure 2).

The first fish individual showing clinical signs (e.g., lethargy at the pond edge) submitted for examination was transported alive in a water bag fed with oxygen on February 2, 2024. The fish was euthanised by a stunning blow to the head, followed by cutting the vertebral column and vessels at the base of the skull. The procedure is in accordance with Czech national legislation, specifically Act No. 246/1992 Coll., on the Protection of Animals against Cruelty, as amended. After that, an examination was conducted and revealed a fish in good nutritional condition, with an increased amount of mucus on the gills but no other gross pathology. Microscopic examination for parasites, using light microscopy of wet mounts scraped from the gills and body surface and squashed tissue preparations, revealed sporadic *Dactylogyrus* sp. monogeneans on the gills, and one and four *Diplostomum* sp. metacercaria in the eyes. Three *Khawia* sp. tapeworms were present in the intestine. Samples for bacteriological testing were obtained from the spleen and kidneys during necropsy. These samples were directly inoculated onto blood agar (Oxoid, UK) and Tryptone Yeast Extract Agar for better detection of flavobacteria (Sigma-Aldrich, USA). Following inoculation, the agar plates were kept at 18°C for 2–5 days and regularly checked for bacterial colony growth. No fish pathogenic germs were cultured. As we suspected CEV infection in this case, a gill sample was processed to identify the virus. DNA was extracted from the gill tissue using the E.Z.N.A.^®^ Tissue DNA Kit (Omega Bio-Tek, UK), following the manufacturer’s instructions. DNA extracts were tested for presence of CEV using nested PCR and real-time PCR, as previously described (15, 19). However, the CEV test for this first carp proved negative (see Table 1).

**Table 1:** Timeline, with dates and results of molecular testing for CEV.

Date	Reason for testing	Result	Water temperature
February 2	First clinical signs without mortality – CEVD?	qPCR negative	2°C
February 21	Differential diagnosis	qPCR positive	6°C
March 6	LAMP/PCR comparison	+/+	9°C
March 27	Virus still present?	LAMP/PCR −/−	14°C

After the ice thawed, clinical signs persisted, with a large proportion of the fish population gathering close to the banks and exhibiting signs of lethargy (Figure 1A and Supplementary Video S1). On February 21, 2024, when water temperatures had reached 6°C, additional gill samples were collected from four fish for further virological testing. To rule out the possibility of ammonia (NH_4_) intoxication in the affected fish (15), we also took blood samples by puncturing the caudal vessels using an 18G needle and examined these using a Konelab 20i biochemical analyzer and commercial test kits (Biovendor, Czech Republic). The results indicated normal levels of blood NH_4_ ranging from 95.0 to 144.5 μmol/L (n = 4). Additionally, we measured water quality parameters using an HQ40D portable multi-meter (Hach, Loveland, Colorado, United States) and standard methods (20). The results indicated that water quality was not the cause of the problem, with pH at 6.97, N-NH_4_ at 0.06 mg/L, N-NO_2_ at 0.034 mg/L, N-NO_3_ at 8.15 mg/L, P-PO_4_ at 0.043 mg/L, and Cl^−^ at 48.60 mg/L.

A second molecular diagnostic examination confirmed that the gill samples from all four fish tested positive for CEV, with Ct values ranging from 22.7 to 29.7. Subsequent Sanger sequencing (SEQme, Czech Republic) of the nested PCR products resulted in an identical sequence for all four samples (GenBank database accession number PQ568129). Phylogenetic sequence analysis then identified the detected agent as belonging to CEV genogroup I.

Fish mortality began to occur gradually 1 week after virus confirmation, as the water temperature increased to 8°C (Figures 1B, C). During this CEV outbreak, we noted a significant difference in mortality rates for Přerov scaly carp (30%) and Hungarian mirror carp M2 (60%) (Difference test between two proportions, *p* < 0.001). Gill samples taken for virological examination for control purposes on March 27, 2024, tested negative for CEV and the outbreak was considered over.

The clinical samples available from this CEV outbreak gave us the opportunity to test the applicability of a novel virus detection method, based on loop-mediated isothermal amplification (LAMP), under field conditions (21). Gill samples of approximately 0.2 cm^3^ were collected directly from four moribund fish on-site on March 6, 2024. As the gill sample required is relatively small, the procedure causes minimal damage to the fish. We also took samples from a fish that had died recently and from a cadaver approximately two-days-old. To assess the effectiveness of environmental DNA (eDNA) detection, we used SVHVL10RC filters with a 0.45 μm pore size and a PVDF membrane with a Luer inlet/outlet (Millipore Sigma, USA). Finally, we obtained three samples of water from the infected pond (2×350 mL and 1×400 ml) using a sterile 25 mL syringe. In each case, we then manually forced the water through the filter until it was fully clogged.

To successfully implement the LAMP method under field conditions, it is essential to have a reliable method of template DNA preparation. Initially, we used 50 μl of PrepManTM Ultra (ThermoFisher Scientific, UK) and boiled it at 100°C for 10 min, as this method would be possible to use in the field. Subsequently, we experimented with boiling the sample at 100°C for 10 min in varying amounts of distilled water (200, 500, and 1,000 μL). In each case, the filters were taken out of their casing, cut into thirds, and then prepared using one of three methods. The first was based on the standard DNeasy Blood & Tissue DNA purification kit (Qiagen, Netherlands), following the manufacturer’s instructions; the second required the filter sample to be boiled at 100°C for 10 min in 200 μL of PrepManTM Ultra; and the third involved boiling the filters in 500 μL of distilled water.

Samples treated with PrepManTM Ultra showed inhibition of amplification, most likely by fish mucus, and required further diluting at least 10x to obtain a positive result. We attempted to improve the results by adding Bovine Serum Albumin to the LAMP mastermix (0.5 μg/μl); however, this had no effect. On the other hand, samples isolated directly into 500 and 1,000 μL of water showed LAMP results comparable with those obtained from diluted PrepManTM Ultra. This allowed us to conduct simple and reliable isolation, at minimal expense, directly in the field. The filters isolated by the kit failed to yield positive results in the subsequent LAMP test, but both the PrepManTM Ultra and water-boiled filters showed positive outcomes. This is unsurprising as the amount of eDNA in the sample was very small and partly lost during the kit isolation procedure. However, positive results from other template preparation methods confirm that eDNA could be considered a viable tool for disease detection in ponds.

Our LAMP procedure was based on primers targeting the *p4A* gene designed by Cano et al. (22), in concentrations recommended by the authors, and use of the portable OptiGene Genie II Isothermal DNA/RNA amplification platform (OptiGene, UK). The mixture contained 10 μL of ISO-004 mastermix (OptiGene, UK), 5 μL of primer mix, and 5 μL of sample. The program was set at 65°C for 40 min, followed by annealing curve analysis between 70–90°C. Positive samples had an amplification peak time below 20 min and an annealing temperature peak between 81.0–81.8°C. To test the sensitivity of the method we used a positive sample prepared by PrepManTM isolation from a carp gill with CEV previously confirmed by qPCR (19). From this positive control sample, we made 10-fold serial dilutions (10^0^–10^−6^) and tested these using the LAMP procedure. Reliable detection of CEV was achieved down to 10^−5^ sample dilution.

The samples from the four living moribund specimens, both the fresh cadaver and the cadaver in a progressed state of decay, and all pond water samples tested positive by LAMP. The samples taken from the fish cadavers displayed annealing temperatures and amplification peak times like those for the live animals. Samples that tested positive were subsequently confirmed by qPCR, thus demonstrating the reliability of the field LAMP method.

## 3 Discussion

Here, we report on a natural CEV infection in overwintering common carp broodstock that first manifested clinically at an unusually low water temperature. Four important take-aways arise from our results.

First, the measured water temperature of 2°C was lower than any previously reported in a pond affected by CEV (23). CEV is known to manifest in deep winter, however, and in our previous study focused on CEV disease pathophysiology, we sampled fish at a fish farm with a water temperature of 4.2°C in mid-December, and all infected fish at the farm were dead by the end of January (15). In temperate regions, the density of freshwater is highest at ca. 4°C, meaning that the denser water sinks as the surface temperature of a freshwater body cools to 4°C (18). This basic principle of thermal stratification means that when the water temperature measured under the ice was 2°C, overwintering carp lying in torpor on the bottom of the pond were exposed to an ambient temperature of 4°C. We may also hypothesize that water column mixing, which occurs at an environmental temperature of 4°C, affects pathogen distribution, possibly bringing the incoming virus closer to the overwintering fish. Likewise, as fish immune system functions are temperature-dependent (6, 23), poorer immune competence at very low water temperatures may determine the outcome of CEV infection, as shown in cases of 100% winter mortality of carp (15). Rapid temperature shifts may also compromise the quality of fish immune responses to pathogen challenges (6). In our case, a period of cold stress in January 2024 was followed by warming temperatures, which appeared to contribute to the outbreak of the disease (Figure 2). Our data concerning the progression of the disease indicate that very low pond water temperatures allowed for the establishment of a permissive CEV infection and that innate immunity, on which carp immune competence mainly depends at these ambient temperatures (6), cleared the infection in surviving fish. Outbreaks of CEV infection are reported to occur following carp translocation and/or introduction of infected fish (14), which is, however, not the case here. As temperature is a key abiotic predisposing factor, stress of translocation and temperature fluctuations probably play a role in the onset of the disease (15).

Second, there were significant differences in mortality rates between overwintering scaly and scaleless carp strains. Different CEV genogroups are known to exhibit variation in virulence and host range (24). CEV primarily infects common carp and koi carp, and while other fish species test positive with low virus loads, true replication has yet to be confirmed (12). To examine the susceptibility of different carp strains to CEV, Adamek et al. (24) experimentally challenged naïve fish with infected carp using the cohabitation method, finding that wild Amur carp, an ancient strain derived from *Cyprinus carpio haematopterus*, were more resistant to infection and failed to develop clinical signs of the disease, unlike Přerov scaly carp, which showed lethargic behavior 6–10 days post-exposure. While both naturally infected common carp strains in our study originate from the subspecies *Cyprinus carpio*, Hungarian mirror carp M2, affected with a 60% mortality rate, were significantly more susceptible than Přerov scaly carp (30% mortality). These findings are similar to those obtained in a second study on the sensitivity of common carp strains and crossbreeds to the infectious agent KHV (*Cyvirus cyprinidallo 3*) (25).

Third, our initial test for CEV proved negative, indicating that molecular detection is infection progression dependent. We hypothesize that, while the low temperature of 4°C facilitated CEV replication and infection on the overwintering hosts, abnormal behavior during the initial stage of the outbreak resulted in carp movement into a water column compartment with temperatures closer to 2°C, reducing the virus’s activity and load. To the best of our knowledge, the effect of water temperatures below 6°C on the course of CEV infection and the pathogen’s interaction with the immune system of overwintering fish has not yet been explored in infection trials. Additionally, studies showing significant viral loads in the tissues of infected fish in the early stages of infection have mostly been undertaken using the CEV genogroup II (23). These factors could possibly affect the rates of CEV distribution in the organism. Nevertheless, the absence of CEV DNA in fish sampled at 2°C, despite displaying apparent clinical signs of CEV, poses a significant diagnostic challenge. Owing to the perceived high cost of broodstock fish by the farmer, only one animal was sacrificed for the examination; however, for virological examination, sampling of ten or more fish is generally recommended (26). The use of gill tissue biopsy applied in later sampling events, rather than lethal methods of sampling, could prove beneficial in cases with high value animals, as well as increasing the probability of a correct diagnosis (27). Similarly, samples taken around 2 months after the outbreak also tested negative for the virus. As CEV does not persist in carp that survive the infection (24), this would explain the absence of CEV DNA as the outbreak waned. Alongside standard methods, we successfully applied the LAMP method for CEV detection and established a sample preparation procedure that would be applicable under field conditions. This now means that suspect CEV cases can be confirmed directly on-site. Note, however, that the method is still under development and that further optimization, testing, and comparison with PCR and qPCR methods is in progress. The sensitivity of the LAMP method, as stated in the original article, provides 10^3^ viral copies in under 25 min (22), while the sensitivity of qPCR is 10^1^ copies (28). This may lead to very weak signals being recognized as negative by LAMP and as marginally positive by qPCR.

Fourth, our results confirm that eDNA methods using specific primers can be employed to detect virus pathogens that pose a threat to freshwater aquaculture, allowing for identification of viruses present in a particular water body without the necessity of conducting invasive fish sampling (29). Use of an eDNA monitoring approach could provide early warning of virus infection outbreaks, allowing timely intervention and proactive management of aquacultural facilities by enhancing biosecurity measures. Furthermore, water samples can be collected from multiple locations, providing a comprehensive overview of virus distribution in the aquatic ecosystem. However, eDNA can be influenced by many environmental factors and can degrade over time, decreasing detection reliability over extended periods. For example, seasonal variation in thermal stratification and water column turnover could influence vertical eDNA distribution and the rate of nucleic acid degradation, which may take up to 7 days in the epilimnion (29, 30). Limitations of the method also include a lack of data on CEV shedding rates, survival, and DNA stability in the aquatic environment, as well as dilution effects specific to the size and flow-through of particular water bodies (31). Importantly, genetic material is also known to persist longer as sedimentary eDNA (32). While virus loads in water could be considered a water quality parameter (33), present methods cannot distinguish between live infectious and inactivated virus particles (34). While ensuring high sensitivity and specificity in virus detection remains a challenge, the LAMP method used in the present case report appears to provide an important and efficient candidate.

To conclude, mass mortality events in valuable common carp broodstock cause significant stress for both farmers and veterinarians called to assist, highlighting the need for timely diagnosis using reliable advanced diagnostic methods for pathogen detection that are applicable on-site. The fish producing industry faces significant challenges due to CEV infection outbreaks, including economic losses and management complications. Future research should focus on understanding the interplay between water temperature and the virulence and dynamics of CEV replication, exploring host responses, and developing effective management strategies to maintain the sustainability of aquafarming.

## Data Availability

The datasets presented in this study can be found in online repositories. The names of the repository/repositories and accession number(s) can be found at: https://www.ncbi.nlm.nih.gov/genbank/, PQ568129.

## Supplementary material

Supplementary Video S1 Fish affected by Carp Edema Virus were lethargic, exhibited abnormal behavior, and gathered close to the bank.

## References

[1] Pörtner HO Farrell AP. Physiology and climate change. Science. (2008) 322:690–2. doi: 10.1126/science.1163156, PMID: 18974339

[2] Brander KM. Global fish production and climate change. Proc Natl Acad Sci USA. (2007) 104:19709–14. doi: 10.1073/pnas.0702059104, PMID: 18077405 PMC2148362

[3] Ficke AD Myrick CA Hansen LJ. Potential impacts of global climate change on freshwater fisheries. Rev Fish Biol Fisher. (2007) 17:581–613. doi: 10.1007/s11160-007-9059-5

[4] Marcos-López M Gale P Oidtmann BC Peeler E. Assessing the impact of climate change on disease emergence in freshwater fish in the United Kingdom. Transbound Emerg Dis. (2010) 57:293–304. doi: 10.1111/j.1865-1682.2010.01150.x, PMID: 20561287

[5] Bowden TJ Thompson KD Morgan AL Gratacap RML Nikoskelainen S. Seasonal variation and the immune response: a fish perspective. Fish Shellfish Immun. (2007) 22:695–706. doi: 10.1016/j.fsi.2006.08.016, PMID: 17116408

[6] Scharsack JP Franke F. Temperature effects on teleost immunity in the light of climate change. J Fish Biol. (2022) 101:780–96. doi: 10.1111/jfb.15163, PMID: 35833710

[7] Karvonen A Rintamäki P Jokela J Valtonen ET. Increasing water temperature and disease risks in aquatic systems: climate change increases the risk of some, but not all, diseases. Int J Parasitol. (2010) 40:1483–8. doi: 10.1016/j.ijpara.2010.04.015, PMID: 20580904

[8] Lõhmus M Björklund M. Climate change: what will it do to fish—parasite interactions? Biol J Linn Soc. (2015) 116:397–411. doi: 10.1111/bij.12584

[9] Panicz R Całka B Cubillo A Ferreira JG Guilder J Kay S. Impact of climate-driven temperature increase on inland aquaculture: application to land-based production of common carp (*Cyprinus carpio* L.). Transbound Emerg Dis. (2022) 69:e2341–50. doi: 10.1111/tbed.14577, PMID: 35488872

[10] Way K Haenen O Stone D Adamek M Bergmann S Bigarré L. Emergence of carp edema virus (CEV) and its significance to European common carp and koi *Cyprinus carpio*. Dis Aquat Org. (2017) 126:155–66. doi: 10.3354/dao03164, PMID: 29044045

[11] Abdelsalam EEE Piačková V. Carp edema virus: host selection and interaction, and potential factors affecting its introduction to the common carp population, distribution, and survival. Aquaculture. (2023) 563:739009. doi: 10.1016/j.aquaculture.2022.739009

[12] Adamek M Heling M Bauer J Teitge F Bergmann SM Kleingeld DW. It is everywhere—a survey on the presence of carp edema virus in carp populations in Germany. Transbound Emerg Dis. (2022) 69:2227–41. doi: 10.1111/tbed.14225, PMID: 34231974

[13] Jung-Schroers V Adamek M Teitge F Hellmann J Bergmann SM Schütze H. Another potential carp killer?: carp edema virus disease in Germany. BMC Vet Res. (2015) 11:114. doi: 10.1186/s12917-015-0424-7, PMID: 25976542 PMC4431602

[14] Lewisch E Gorgoglione B Way K El-Matbouli M. Carp edema virus/koi sleepy disease: an emerging disease in central-East Europe. Transbound Emerg Dis. (2015) 62:6–12. doi: 10.1111/tbed.12293, PMID: 25382453

[15] Pikula J Pojezdal L Papezikova I Minarova H Mikulikova I Bandouchova H. Carp edema virus infection is associated with severe metabolic disturbance in fish. Front Vet Sci. (2021) 8:679970. doi: 10.3389/fvets.2021.679970, PMID: 34095283 PMC8169968

[16] Papežíková I Piačková V Dyková I Baloch AA Kroupová HK Zusková E. Clinical and laboratory parameters of carp edema virus disease: a case report. Viruses Basel. (2023) 15:1044. doi: 10.3390/v15051044, PMID: 37243132 PMC10221307

[17] Palíková M Pojezdal Ľ Dávidová-Geržová L Nováková V Pikula J Papežíková I. Carp edema virus infection associated gill pathobiome: a case report. J Fish Dis. (2022) 45:1409–17. doi: 10.1111/jfd.13670, PMID: 35708022

[18] Wetzel RG. Limnology: Lake and river ecosystems. London: Academic Press (2001).

[19] Matras M Borzym E Stone D Way K Stachnik M Maj-Paluch J. Carp edema virus in polish aquaculture – evidence of significant sequence divergence and a new lineage in common carp *Cyprinus carpio* (L.). J Fish Dis. (2017) 40:319–25. doi: 10.1111/jfd.12518, PMID: 27453481

[20] APHA. Standard methods for the examination of water and wastewater. 20th ed. Washington DC: American Public Health Association, American Water Works Association and Water Environmental Federation (1998).

[21] Adams A Thompson K. Recent applications of biotechnology to novel diagnostics for aquatic animals. Rev Sci Tech Off Int Epiz. (2008) 27:197–209. doi: 10.20506/rst.27.1.1792 18666488

[22] Cano I Worswick J Mulhearn B Stone D Wood G Savage J. A seasonal study of koi herpesvirus and koi sleepy disease outbreaks in the United Kingdom in 2018 using a pond-side test. Animals. (2021) 11:459. doi: 10.3390/ani11020459, PMID: 33572469 PMC7916346

[23] Machat R Pojezdal L Piackova V Faldyna M. Carp edema virus and immune response in carp (*Cyprinus carpio*): current knowledge. J Fish Dis. (2021) 44:371–8. doi: 10.1111/jfd.13335, PMID: 33460151

[24] Adamek M Oschilewski A Wohlsein P Jung-Schroers V Teitge F Dawson A. Experimental infections of different carp strains with the carp edema virus (CEV) give insights into the infection biology of the virus and indicate possible solutions to problems caused by koi sleepy disease (KSD) in carp aquaculture. Vet Res. (2017) 48:12. doi: 10.1186/s13567-017-0416-7, PMID: 28222784 PMC5320791

[25] Piačková V Flajšhans M Pokorová D Reschová S Gela D Čížek A. Sensitivity of common carp, *Cyprinus carpio* L., strains and crossbreeds reared in the Czech Republic to infection by cyprinid herpesvirus 3 (CyHV-3; KHV). J Fish Dis. (2013) 36:75–80. doi: 10.1111/jfd.12007, PMID: 23009156

[26] WOAH. Manual of diagnostic tests for aquatic animals: Infection with koi herpesvirus. Paris: Office International des Epizooties (2024).

[27] Matras M Stachnik M Borzym E Maj-Paluch J Reichert M. Distribution of carp edema virus in organs of infected juvenile common carp. J Vet Res. (2023) 67:333–7. doi: 10.2478/jvetres-2023-0049, PMID: 37786850 PMC10541666

[28] Adamek M Matras M Jung-Schroers V Teitge F Heling M Bergmann SM. Comparison of PCR methods for the detection of genetic variants of carp edema virus. Dis Aquat Org. (2017) 126:75–81. doi: 10.3354/dao03152, PMID: 28930088

[29] Bohara K Yadav AK Joshi P. Detection of fish pathogens in freshwater aquaculture using eDNA methods. Diversity. (2022) 14:1015. doi: 10.3390/d14121015

[30] Littlefair JE Hrenchuk LE Blanchfield PJ Rennie MD Cristescu ME. Thermal stratification and fish thermal preference explain vertical eDNA distributions in lakes. Mol Ecol. (2021) 30:3083–96. doi: 10.1111/mec.15623, PMID: 32888228

[31] Longshaw M Feist S Oidtmann B Stone D. Applicability of sampling environmental DNA for aquatic diseases. Bull Eur Assoc Fish Pathol. (2012) 32:69–76.

[32] Turner CR Uy KL Everhart RC. Fish environmental DNA is more concentrated in aquatic sediments than surface water. Biol Conserv. (2015) 183:93–102. doi: 10.1016/j.biocon.2014.11.017

[33] Gomes GB Hutson KS Domingos JA Chung C Hayward S Miller TL. Use of environmental DNA (eDNA) and water quality data to predict protozoan parasites outbreaks in fish farms. Aquaculture. (2017) 479:467–73. doi: 10.1016/j.aquaculture.2017.06.021

[34] Bohmann K Evans A Gilbert MTP Carvalho GR Creer S Knapp M. Environmental DNA for wildlife biology and biodiversity monitoring. Trends Ecol Evol. (2014) 29:358–67. doi: 10.1016/j.tree.2014.04.003 24821515

